# Progesterone Actions During Central Nervous System Development

**DOI:** 10.3389/fnins.2019.00503

**Published:** 2019-05-17

**Authors:** Juan Carlos González-Orozco, Ignacio Camacho-Arroyo

**Affiliations:** Unidad de Investigación en Reproducción Humana, Instituto Nacional de Perinatología–Facultad de Química, Universidad Nacional Autónoma de México, Mexico City, Mexico

**Keywords:** progesterone, progesterone receptor, neurodevelopment, neuroprotection, myelination, brain sex differentiation, brain tumors

## Abstract

Although progesterone is a steroid hormone mainly associated with female reproductive functions, such as uterine receptivity and maintenance of pregnancy, accumulating data have shown its physiological actions to extend to several non-reproductive functions in the central nervous system (CNS) both in males and females. In fact, progesterone is *de novo* synthesized in specific brain regions by neurons and glial cells and is involved in the regulation of various molecular and cellular processes underlying myelination, neuroprotection, neuromodulation, learning and memory, and mood. Furthermore, progesterone has been reported to be implicated in critical developmental events, such as cell differentiation and neural circuits formation. This view is supported by the increase in progesterone synthesis observed during pregnancy in both the placenta and the fetal brain. In the present review, we will focus on progesterone actions during CNS development.

## Introduction

Progesterone (Pregn-4-ene-3,20-dione) is a sex steroid hormone classically associated with female reproductive functions, including sexual behavior, uterus preparation for embryo implantation and maintenance of pregnancy (Wu et al., 2018). Although this hormone is mainly synthesized in the ovaries and placenta, it is also produced by the adrenal cortex as well as the central nervous system (CNS) of both male and female mammals (Gutai et al., 1977; Mensah-Nyagan et al., 1999; Tuckey, 2005). In addition, females of several species present higher progesterone levels in their circulating plasma than males (Tuckey, 2005; Lauretta et al., 2018). However, the fact that both males and females synthesize this hormone indicates that its functions are not limited to the female reproductive physiology. For example, progesterone regulates various non-reproductive functions in the CNS related to neuroprotection, neuromodulation, myelination, neurogenesis, neuronal plasticity, and mood (Snyder and Hull, 1980; Schumacher et al., 2004). Therefore, given that progesterone is synthesized, metabolized and exerts its actions in the CNS, it is referred to as a neurosteroid.

Progesterone synthesis and actions in the CNS are reported in several vertebrate species. Specifically, neurons and glial cells in the brain can synthesize it *de novo* from cholesterol as they express the enzymes responsible for its synthesis and metabolism (Testas et al., 1989; Mellon and Deschepper, 1993; Schumacher et al., 2004). Thereafter, the progesterone resulting from either circulating plasma or CNS local synthesis binds to its specific intracellular and membrane receptors to regulate the molecular and cellular processes underlying the brain functions. Furthermore, accumulating data suggest the progesterone actions in the CNS not to be restricted to the adult life and to be present since fetal life during neural development in both genders (Schumacher et al., 2014).

Existing evidence indicates the participation of progesterone in some key events, such as neurogenesis, neuroprotection, neural circuit organization, oligodendrogenesis, myelination, and brain sex differentiation. Remarkably, pregnancy is characterized by an increase in progesterone levels both in the maternal plasma and the fetal circulation. In fact, the enzymes responsible for progesterone synthesis and the progesterone receptors were observed to be expressed early in the fetal life in several species, including chicks, rodents, sheep and humans (Milewich et al., 1991; Ukena et al., 1999; Camacho-Arroyo et al., 2003; Nguyen et al., 2003). Therefore, progesterone was suggested to have a fundamental role in the maternal and fetal brain adaptation during pregnancy, as well as later during critical CNS developmental events (Pluchino et al., 2016).

In this review, we summarize the implications of progesterone in the molecular and cellular processes underlying CNS development. In addition, given that the carcinogenesis processes often recapitulate developmental programs, some insights into the possible participation of progesterone in CNS tumors development are provided at the end of the review.

## Progesterone Synthesis and Its Mechanisms of Action

### Synthesis and Sources of Progesterone in the Developing CNS

In vertebrates, cholesterol is the common precursor for progesterone biosynthesis. Specifically, it is taken up by steroidogenic cells in the endocrine tissues (mainly the ovaries in females and the adrenal glands in males) from the blood plasma, where it is transported as low-density lipoprotein (LDL) cholesterol and is internalized by receptor-mediated endocytosis in vesicles. These are then fused to lysosomes to allow the release of the free cholesterol form present in the cytoplasm (Vickery, 1993; Schumacher et al., 2004) and start the process of steroidogenesis.

Importantly, cholesterol is also synthesized *de novo* by steroidogenic cells through the condensation of two molecules of acetyl-CoA, forming acetoacetyl-CoA which is successively converted into 3-hydroxy-3-methylglutaryl-CoA (HMG-CoA). Thereafter, the HMG-CoA reductase enzyme converts HMG-CoA to mevalonate, which is used to produce the two cholesterol precursors, i.e., squalene and lanosterol, by the squalene synthase and lanosterol synthase enzymes, respectively (Pasqualini and Chetrite, 2016).

Similarly, the CNS is an active site of cholesterol synthesis, given that the blood-brain barrier is not permeable to LDL cholesterol. Therefore, all the cholesterol present in the CNS derives from its local synthesis by neurons and glial cells (astrocytes and oligodendrocytes) (Schumacher et al., 2004). This capacity of the CNS to synthesize cholesterol is preserved from prenatal development in various mammalian species, including the mouse, guinea pig, sheep and humans (Dietschy, 2009).

**FIGURE 1:**
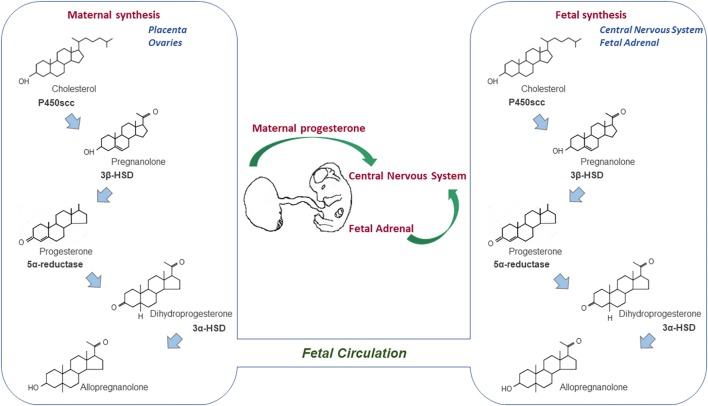
Sources of progesterone for the developing CNS. Progesterone synthesis begins with the conversion of cholesterol into pregnenolone by the cytochrome P450scc, which is in turn converted into progesterone by the 3β-HSD enzyme. Thereafter, the 5α-reductase enzyme can metabolize progesterone to 5α-dihydroprogesterone, which is further reduced to allopregnanolone. Progesterone synthesized by the placenta (ovaries in rodents) during pregnancy crosses the placental barrier, enters the fetal circulation and reaches the developing CNS. However, the fetal adrenal glands and CNS are also locations in which progesterone is synthesized and metabolized during the prenatal life.

The progesterone synthesis presents a conserved pathway in the vertebrate species, which begins with the transport of accumulated cholesterol from the outer to the inner mitochondrial membranes by transporter proteins, such as the 18 kDa translocator protein (TSPO) and the steroidogenic acute regulatory protein (StAR) (Sierra, 2004; Papadopoulos et al., 2006). Thereafter, cholesterol is converted to pregnenolone in the inner mitochondrial membrane by the cytochrome P450scc, which is then converted to progesterone in both the mitochondria and cytoplasm by 3β-hydroxysteroid dehydrogenase (3β-HSD). The newly synthesized progesterone can either exert its physiological effects in an autocrine and paracrine manner (Chaffkin et al., 1992; Schumacher et al., 2001), or can be metabolized by the 5α-reductase enzyme to 5α-dihydroprogesterone (5α-DHP), which is further reduced to allopregnanolone (3α,5α-THP) by 3α-hydroxysteroid oxidoreductase (3α-HSD) (Figure 1) (Melcangi et al., 1998). Finally, both metabolites, the 5α-DHP and allopregnanolone are active neurosteroids, with the latter having a relevant role also during CNS development (Schumacher et al., 2014; Pluchino et al., 2016).

Given its lipophilic structure, the progesterone produced from steroidogenic tissues, including the gonads and the adrenal glands, can cross the blood–brain barrier to reach the CNS. Importantly, the enzymes necessary for the synthesis and metabolism of progesterone are also expressed in neuronal and glial lineages in the adult CNS, in a region and cell-dependent manner (Testas et al., 1989; Mellon and Deschepper, 1993; Schumacher et al., 2004). However, the presence of these enzymes is not restricted to the adult life, as both their expression and activity are also found during the early ages of the mammalian neural development (Compagnone and Mellon, 2000).

Progesterone’s potential actions throughout development are supported by the high concentrations of the hormone during mammalian pregnancy in both the maternal and fetal circulation (Morel et al., 2016). For example, the female and male fetal plasma concentrations of progesterone and its metabolite allopregnanolone were found to increase throughout pregnancy in the ungulate species (e.g., sheep), together with their fetal brain concentrations, especially during late pregnancy (Nguyen et al., 2003). This indicates that both progesterone and allopregnanolone play a significant role during CNS development in mammals.

The developing mammalian fetus is continuously exposed to progesterone during pregnancy. While the fetal ovary does not actively synthesize steroid hormones until birth in rodents (Greco and Payne, 1994), the circulating progesterone levels in developing male and female rodents and sheep are found at equal levels (Weisz and Ward, 1980; Nguyen et al., 2003). Therefore, the potential sources of progesterone for the developing fetus were suggested to be the placenta, maternal ovary (specifically in rodents), fetal adrenal gland, and *de novo* synthesis of progesterone within the developing CNS (Figure 1) (Compagnone and Mellon, 2000; Tuckey, 2005; Morel et al., 2016).

The conversion of cholesterol into pregnanolone by P450scc is the first rate-limiting step in progesterone synthesis. In fact, the P450scc protein expression is detected by immunohistochemistry in the neural crest cells since embryonic day 9.5 (E9.5) in mice and at E10.5 in rats (Compagnone et al., 1995). This expression is maintained throughout fetal development and continues to be detected before birth in structures of the peripheral nervous system (PNS) derived from the neural crest, including the dorsal root ganglia, trigeminal ganglion and retina. Furthermore, mice and rats present P450scc expression in the neurons, astrocytes, and oligodendrocytes of various brain regions (e.g., the cortex, thalamus, hypothalamus, hippocampus and spinal cord) during prenatal life, after birth, and adulthood (Mellon and Deschepper, 1993; Compagnone et al., 1995). In contrast, cerebellar P450scc is only expressed in early postnatal male rats (Ukena et al., 1998).

Similarly, 3β-HSD expression was detected in the CNS during prenatal and early postnatal development in several vertebrates, and particularly, in the mammal species. Interestingly, while 3β-HSD expression and activity were found to significantly increase throughout the brain right before birth and during the first postnatal days in rodents and sheep, without a marked sexual dimorphism, the cerebellum exhibits the highest production of progesterone within the CNS during neonatal life in rats (Ukena et al., 1999; Compagnone and Mellon, 2000; Sakamoto et al., 2001; Nguyen et al., 2003). In this developmental period, the CNS undergoes indispensable developmental processes, such as synaptogenesis, myelination, organization, and remodeling of the neural circuits (Stiles and Jernigan, 2010). Although the expression and activity of 3β-HSD are relevant during postnatal CNS development, enzymatic activity of 3β-HSD was observed as early as the second trimester of pregnancy in the human fetal brain, indicating the progesterone synthesis to be present from prenatal development (Milewich et al., 1991).

With regards to the enzymes involved in progesterone metabolism, the expression and activity of both the 5α-reductase and 3α-HSD were detected in the female and male fetal brain of guinea pig and sheep from the second half of the pregnancy and in late pregnancy in rodents. This leads to high concentrations of allopregnanolone in different brain regions in all the mentioned species during prenatal and the first postnatal days (Compagnone and Mellon, 2000; Nguyen et al., 2003; Kelleher et al., 2011; Hirst et al., 2014). Moreover, both the levels of P450scc and 5α-reductase increase in the CNS in late pregnancy, suggesting that the CNS has its highest capacity for progesterone and allopregnanolone synthesis around the time of birth (Nguyen et al., 2003; Kelleher et al., 2011).

Progesterone is essential for the maintenance of pregnancy and its circulating levels in pregnant women increase between sixfold and eightfold when compared with non-pregnant subjects due to placental secretion (Lee et al., 2017). Existing evidence from pregnant rats indicates that progesterone from the maternal circulation enters the fetal bloodstream and reaches the developing CNS, binding to its intracellular receptors (Wagner and Quadros-Mennella, 2017). Additionally, progesterone from the human placenta was proposed to contribute to adequate neurodevelopment, having specific roles in neuroprotection and the development of neural circuits. This view is supported by the impaired neurodevelopment observed in preterm birth possibly resulting from the premature disruption of the supply of progesterone and another steroid hormone (Trotter et al., 2001), however, more in-depth studies are still needed to confirm it.

### Mechanisms Underlying the Progesterone Action During CNS Development

**FIGURE 2:**
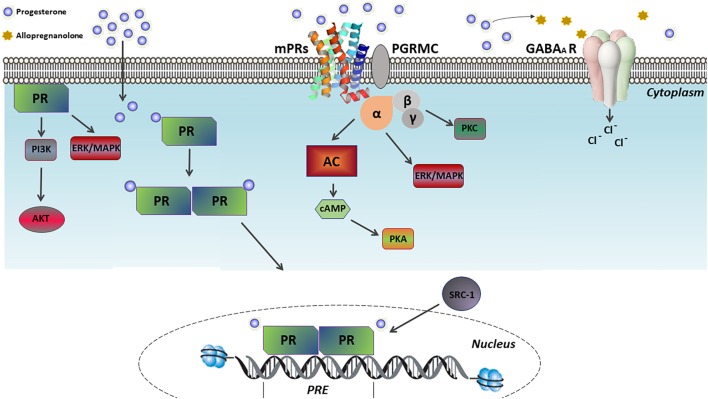
Mechanism of progesterone actions. Progesterone exerts its effects on its target cells through two central pathways, namely the classical and non-classical ones. In the classical pathway, progesterone binds to its intracellular receptor (PR), activating such a transcription factor which dimerizes and translocates to the nucleus. There, it binds to specific DNA sequences, called progesterone response elements (PRE), which are mainly located in the gene promoter regions, thus regulating their expression. In contrast, the non-classical pathway involves the PR ligand-independent activation by membrane-associated kinases and the activation of multiple G protein-coupled membrane receptors of progesterone (mPRs), which in turn activates pathways related to cAMP-dependent protein kinase A (PKA), Ca^2+^-dependent protein kinase C (PKC), PI3K/Akt and ERK/MAPK. Furthermore, the non-classical action of progesterone includes the modulation of the γ-aminobutyric acid type A (GABA-A) receptors following its conversion into allopregnanolone.

Progesterone regulates several reproductive and non-reproductive functions in vertebrates. Specifically, its action in its target cells is promoted by two central pathways, namely the classical and non-classical ones. The classical signaling pathway (also known as the genomic pathway) involves progesterone crossing the plasma membrane due to its lipophilic structure and binding to its intracellular receptor (PR). This leads to the activation of the transcription factor, which dimerizes and translocates to the nucleus. There, it binds to specific DNA sequences [i.e., the progesterone response elements (PRE)] that are mainly located in the gene promoter regions, thus regulating their expression (Figure 2) (Camacho-Arroyo et al., 2002; Liu and Ogle, 2002).

Although two PR isoforms (i.e., PR-A and PR-B) are encoded in the same gene, they have different transcription start sites, resulting in the PR-A being the N-terminal truncated form of the complete PR-B isoform (Kastner et al., 1990; Mesiano et al., 2011). Both isoforms are found in the brain and spinal cord of vertebrate species (Kato and Onouchi, 1997; Kastrup et al., 1999; Labombarda et al., 2002; Guerra-Araiza et al., 2003; Camacho-Arroyo et al., 2018) and, although being structurally very similar, they present a different expression pattern, regulation, and function. In general, given that the PR-B is a more potent transcription activator than the PR-A, they differentially regulate gene expression (Horwitz et al., 1996). Transcriptional functions of the ligand-bound PR isoforms include the recruitment of coactivators (e.g., SRC-1 and CBP), the transcription of corepressors (e.g., the N-CoR1), the consensus sequences PRE, the recruitment of chromatin remodeling complexes (e.g., the SWI/SNF complex), and the integration of the signaling pathways from the cytoplasm, which are in turn influenced by the cellular context and/or developmental stage (Vicent et al., 2006; Kumar et al., 2013; Tetel and Acharya, 2013; Grimm et al., 2016).

On the other hand, the non-classical (or non-genomic) signaling pathway involves several molecular processes related to rapid cellular responses, ranging from minutes to a few hours after progesterone exposure. Examples include the PR ligand-independent activation by membrane-associated kinases, the activation of G protein-coupled membrane progesterone receptors (e.g., mPRα, mPRβ, mPRγ, mPRδ, and mPRε subtypes), the stimulation of the progesterone receptor membrane components (PGRMC1/2) to enhance the activity of mPRs, and the modulation of the gamma-aminobutyric acid type A (GABA-A) receptors after the conversion into allopregnanolone (Figure 2) (Follesa et al., 2001; Boonyaratanakornkit et al., 2008; Valadez-Cosmes et al., 2016). In addition, among the signaling pathways either extra-nuclearly activated through PR ligand-independent stimulation or activated by mPRs, the cAMP-dependent protein kinase A (PKA), Ca^2+^-dependent protein kinase C (PKC), PI3K/Akt and ERK/MAPK pathway can be found (Figure 2) (Zhu et al., 2003; Valadez-Cosmes et al., 2016; Garg et al., 2017).

**FIGURE 3:**
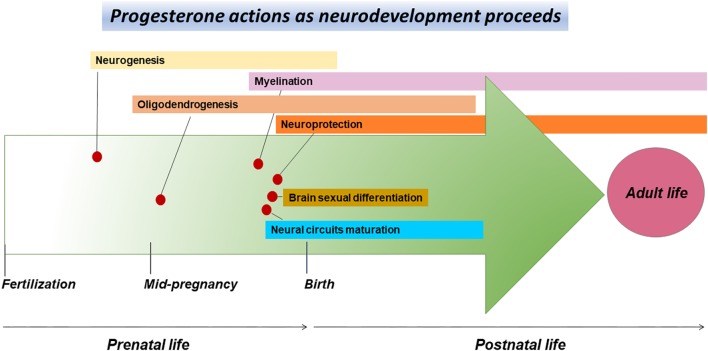
CNS development events associated with the progesterone actions. The arrow of time represents the CNS development course during prenatal and early postnatal life, as well as some of the events associated with the progesterone actions occurring as the neurodevelopment proceeds. These actions include proliferation and differentiation of neural progenitor cells during prenatal neurogenesis, the maturation of oligodendrocytes and myelination before and after birth, neuroprotection before and after birth, sexual differentiation of the brain right before and shortly after birth, as well as the maturation of neural circuits in the late stages of pregnancy and during the first postnatal days.

While PR expression is observed in different brain regions, the expression of its isoforms is regionally and developmentally regulated (Table 1) (Camacho-Arroyo et al., 2003; Wagner, 2008; Palliser et al., 2012). Although both the PR isoforms are expressed in the brain of male and female chicks as early as E8 (Camacho-Arroyo et al., 2003), their developmental expression has been more widely studied in rodents during the prenatal and early postnatal stages. In fact, PR expression was detected by immunohistochemistry in the forebrain regions of E17 rats, which include the amygdala, basal ganglia, thalamus, hippocampus, subventricular zone, and the cerebral cortex. In the latter, PR was observed to be transiently expressed during perinatal rat development. While it reaches elevated levels between E17 and postnatal day 2 (P2), it decreases progressively until day P14, consistently in both genders (Quadros et al., 2007; Jahagirdar and Wagner, 2010), and its expression is maintained during adulthood (Camacho-Arroyo et al., 1998; Guerra-Araiza et al., 2001). Considering that the PR levels specifically increase during a brief period around birth in a time-window critical for brain cortex maturation in rodents, progesterone was indicated to contribute to their adequate cortical development through its classical mechanism of action (Jahagirdar and Wagner, 2010). Furthermore, PR was proposed to play a key role during brain sexual differentiation, since its expression was detected from E18 in most sexually dimorphic regions of the rat brain (e.g., the preoptic area), with a substantial higher expression in males and no detectable expression in females until postnatal day 10 (P10) (Quadros et al., 2002a). Other regions of the developing CNS expressing PR include the late prenatal and early postnatal ventral midbrain of mice and rats of both genders. Therefore, Progesterone was indicated to participate in the development of the nigrostriatal pathway connectivity (Beyer et al., 2002; Quadros et al., 2008) and the early postnatal cerebellum development of rats (Quadros et al., 2008). With regards to the action of progesterone through the non-classical mechanism in the developing CNS, a widely distributed expression of mPRβ was recently observed in the female and male fetal brain and spinal cord of mice starting from E15.5 (Kasubuchi et al., 2017). Progesterone was suggested to contribute to the global development of the CNS and its actions were found to be mediated through both the classical and non-classical pathways. Thus, multiple functions have been associated with the progesterone actions during CNS development (Figure 3), which will be described in the following sections.

**Table 1:** Expression of the progesterone receptors during prenatal and early postnatal neurodevelopment.

Region	Structure	Detected	Experimental	Developmental	Species	References
		receptor	method	stage		
Telencephalon	Cerebrum	PR-A/PR-B	Western blot	E8	Chicken	Camacho-Arroyo et al., 2003
	Cerebrum	PR-A/PR-B	Western blot	E35	Guinea pig	Palliser et al., 2012
	Cerebrum	mPRβ	*In situ* hybridization	E15.5	Mouse	Kasubuchi et al., 2017
	Cortex	Total PR	Immunohistochemistry	E18	Rat	Jahagirdar and Wagner, 2010
	Cortex	PR-A/PR-B	qPCR	E20	Rat	Kato et al., 1993
	Basal ganglia	Total PR	Immunohistochemistry	E17	Rat	Quadros et al., 2007
	Subventricular zone	Total PR	Immunohistochemistry	E17	Rat	Quadros et al., 2007
	Hippocampus	Total PR	Immunohistochemistry	P1	Rat	Quadros et al., 2007
	Amygdala	Total PR	Immunohistochemistry	E18	Rat	Quadros et al., 2007
Diencephalon	Thalamus	Total PR	Immunohistochemistry	E20	Rat	Quadros et al., 2007
	Hypothalamus	Total PR	Immunohistochemistry	E18	Rat	Quadros et al., 2002a
	Hypothalamus	Total PR	Immunohistochemistry	E4	Chicken	Guennoun et al., 1987
	Hypothalamus	PR-A/PR-B	Western blot	P1	Chicken	Camacho-Arroyo et al., 2018
	Pituitary gland	Total PR	Radiolabeling	P1	Rat	MacLusky and McEwen, 1980
Mesencephalon	Midbrain	Total PR	Immunofluorescence	P1	Rat	Willing and Wagner, 2016
	Midbrain	PR-A/PR-B	Western blot	P1	Chicken	Camacho-Arroyo et al., 2018
Metencephalon	Cerebellum	Total PR	Immunohistochemistry	P1	Rat	Quadros et al., 2008
	Cerebellum	PR-A/PR-B	Western blot	P1	Chicken	Camacho-Arroyo et al., 2018
Spinal cord	Spinal cord	mPRβ	*In situ* hybridization	E15.5	Mouse	Kasubuchi et al., 2017


## Progesterone Actions During CNS Development

### Progesterone Effects on Neural Progenitor Cells Proliferation and Neuronal Differentiation

Neurogenesis refers to the generation of the distinct neurons of the nervous system from neural progenitor cells. Progesterone actions on the neural precursors (Figure 3) were hypothesized given that early neural progenitor cells derive from the neurogenic ventricular and subventricular zones of rats, which are known to express the polysialylated-neural cell adhesion molecule (PSA-NCAM) that can synthesize and metabolize progesterone (Gago et al., 2001). PSA-NCAM progenitor cells conserve both a neurogenic and gliogenic potential of differentiation, which mainly depends on the stimuli they receive. Progesterone and allopregnanolone were shown to promote the proliferation of PSA-NCAM progenitors isolated from the brain of newborn rats, in a concentration-dependent manner and without differences between genders (Gago et al., 2004). Moreover, allopregnanolone regulates cell proliferation in the developing cortex of rats by modulating the activity of GABA-A receptors during the early neonatal life of rodents (Grobin et al., 2006). In addition, progesterone was reported to regulate the proliferation of neural progenitor cells in the subgranular zone of the hippocampal dentate gyrus of sheep and rats (Yawno et al., 2009; Barha et al., 2011; Bali et al., 2012) which is, along with the subventricular zone of the lateral ventricles, the only site in the CNS to retain a neurogenic potential during adulthood.

The effects of progesterone on the neuronal differentiation were also investigated in dopaminergic neurogenesis. *In vitro* studies described progesterone to increase the number of differentiated dopaminergic neurons and the expression of the dopamine transporter in the neural progenitors to derive from mice embryonic cells. This result is consistent with additional *in vivo* studies demonstrating abnormalities in the differentiation of dopaminergic neurons in PR knockout mice (Woolley et al., 2006; Díaz et al., 2007, 2009), highlighting the role of progesterone in dopaminergic neurogenesis and the establishment of the dopaminergic pathways during development through the classical mechanism of action (Figure 2). Further studies should be performed to identify the mechanisms involved in neuronal differentiation which are promoted by progesterone.

### The Role of Progesterone in Myelination During CNS Development

Positive effects of progesterone on nerve myelination have been documented in mammals, starting with its vital role in promoting the myelination of peripheral nerves damaged after traumatic injury in rodent models (Schumacher et al., 2001). These observations were later expanded to the CNS (Ghoumari et al., 2003), leading to the proposal of the therapeutic use of progesterone for the treatment of demyelinating lesions and diseases in humans (Schumacher et al., 2012). Moreover, progesterone was suggested to be necessary for nerve myelination during the mammalian development. While myelination begins in the CNS during the fetal life at late gestation in humans, it mainly occurs just before birth in rodents (Figure 3) (Barateiro and Fernandes, 2014). Using organotypic cerebellum cultures obtained from postnatal rats and mice, progesterone was demonstrated to stimulate myelination by inducing the proliferation of oligodendrocyte progenitor cells (OPCs), promoting their differentiation into oligodendrocytes (the myelinating cells of the CNS), and finally by accelerating myelin synthesis within these cells, as evidenced by the increase in myelin basic protein (MBP) expression. These actions are known to be mediated by PR activation (Figure 2), given that they were inhibited by mifepristone and promoted by the PR selective agonist R5020, similarly to progesterone uniformly between genders (Ghoumari et al., 2003, 2005).

Furthermore, progesterone may promote myelination through its conversion into allopregnanolone, as the administration of finasteride to pregnant guinea pigs reduces myelination (measured by MBP expression) in the subcortical white matter of the offspring. Moreover, the blockage of progesterone and other steroid hormones flow from the maternal placenta to the fetus at mid-pregnancy decreases MBP expression in their fetal brain. In these experiments, PR expression is upregulated due to a reduction in progesterone levels, an effect which was found to be higher in females (Palliser et al., 2012). Observations of progesterone actions in myelination are also corroborated by *in vitro* studies using organotypic cerebellum cultures from postnatal male mice exposed to lysophosphatidylcholine (LPC) as a model of demyelination. Progesterone treatment significantly enhanced myelination of axons (seen by MBP immunostaining) when compared to the non-treated cerebellar cultures and the PR *knockout* mice-derived cerebellar cultures (Hussain et al., 2011). The importance of progesterone and its metabolites in oligodendrocyte differentiation and myelination is also supported by the fact that rodents’ OPC and mature oligodendrocytes actively synthesize and metabolize progesterone throughout the myelin formation process (Gago et al., 2001).

Finally, progesterone was described to stimulate both OPC proliferation and oligodendrocyte differentiation in the adult CNS of rats, especially after demyelinating damage (Labombarda et al., 2009). This is relevant given that the remnant OPC found in the adult CNS are produced early in life during the CNS development, indicating that these cells retain their ability to respond to progesterone throughout life.

### Organizational Actions of Progesterone During Neurodevelopment

Neurons and glial cells form complex functional networks implicated in several functions carried out by the CNS. Although the arrangement of these networks, usually referred to as neural circuits, varies according to the related function of each CNS region (LoTurco, 2000), the processes that contribute to the formation of such neural circuits are similar throughout. The general mechanisms that shape the neural circuitry occur late in neurodevelopment (Figure 3) and include neuronal maturation, axons and dendrites arborization, axon guidance supported by glial cells, the establishment of synaptic connections between neurons and the elimination of improper connections (Weiner et al., 2013). Progesterone contributes to the organization and the establishment of neural circuits during mammal development by promoting cell maturation, dendrites formation, synaptogenesis and axonal myelination, as documented in rats and guinea pigs (Sakamoto et al., 2001; Tsutsui, 2008; Palliser et al., 2012).

The organizational effects of progesterone were mainly investigated in the developing cerebellum of rats. In fact, the expression of the cytochrome P450scc enzyme is immediately detected in this region after Purkinje neuron differentiation, whereas the expression of the 3β-HSD enzyme progressively increases after birth, accompanied with an enhancement of progesterone synthesis by Purkinje neurons (Ukena et al., 1998, 1999) in a critical period for the neural circuit formation in the cerebellar cortex. Using cerebellar slice cultures from newborn male rats, progesterone was identified to increase the dendritic growth and dendritic spine formation, in a dose-dependent manner, through its interaction with the PR (Figure 2). In contrast, the synaptic density of Purkinje neurons is enhanced *in vivo* when male newborn rats are treated with exogenous progesterone, as observed by electron microscopy (Sakamoto et al., 2001, 2002). This suggests that endogenous progesterone synthesized *de novo* by rat Purkinje neurons actively contributes to the formation of the cerebellar neural circuit after birth (Sakamoto et al., 2002), promoting Purkinje dendritic growth and synaptogenesis by upregulating the expression of neurotrophic factors involved in such processes [i.e., brain-derived neurotrophic factor (BDNF) and neurotrophin-3 (NTF3)] (Tsutsui et al., 2011).

Furthermore, progesterone was also proposed to influence the formation of cortical neural circuits. PR expression is developmentally regulated in the cerebral cortex and has been detected as early as E18 in the rats’ subplate, a layer of cortical neurons indispensable for cerebral cortex maturation and circuitry organization. PR expression within the postnatal cortex peaks at age P2, after which it progressively declines until P14. This period of high PR expression coincides with a critical period of cortical development and connectivity establishment in rats (Jahagirdar and Wagner, 2010). In addition, while PR expression is detected in pyramidal neurons of the cortical layer V as well as in cortical layers II and III of P3 and P6 rats, respectively, reaching a peak of expression between P7 and P10, it later declines until P27 (Quadros et al., 2007; Lopez and Wagner, 2009). These observations indicate that PR expression is relevant for normal cortical development, as supported by a study with PR *knockout* mice model, in which males and females reported abnormalities in their somatosensory-dependent motor reflexes due to incorrect sensorimotor cortex development (Willing and Wagner, 2014). Similarly, another study indicated that the administration of exogenous progesterone to pregnant rats increases dendritic branching and the number of dendritic spines in the pyramidal neurons of the somatosensory cortex of the offspring (Menzies et al., 1982), highlighting the crucial role of PR activation by progesterone in the formation of cortical neural circuits.

A similar pattern of PR expression is also observed in the developing mesocortical (dopaminergic) pathway of mice and rats, which projects from the midbrain ventral tegmental area (VTA) to the prefrontal cortex. In both species, PR expression is high during the first postnatal week and then declines in both genders. Administration of mifepristone during this postnatal period decreases both the tyrosine hydroxylase expression in the VTA and the dopaminergic innervation of the prelimbic prefrontal cortex (especially in male rats), impairing cognitive behaviors later in adulthood (Beyer et al., 2002; Quadros et al., 2008; Willing and Wagner, 2016). These data suggest that progesterone also participates in the adequate establishment of the neural circuits of the mesocortical dopaminergic pathway.

### Progesterone Effects on Brain Sexual Differentiation

Given the significant differences in PR expression in several brain regions between male and female vertebrates at critical stages of neurodevelopment progesterone was proposed to be involved in brain sexual differentiation.

Differences in prenatal and postnatal PR expression between male and female rats are observed in sex dimorphic brain regions, such as the medial preoptic nucleus (MPN) of the preoptic area, the arcuate nucleus, and the paraventricular nucleus (PVN) of the hypothalamus. While the PR expression in these regions is detectable at the mRNA and protein levels in males from E18, it is only observed in females from P10 (Quadros et al., 2002a, 2007). Sex differences in dimorphic regions are also noticed in PR isoforms expression in the brain of newborn chicks. In fact, females’ hypothalamus presents a higher content of PR-A when compared to that of males at postnatal age P1 (Camacho-Arroyo et al., 2018).

The observed differences in PR expression indicate the existence of a period during neurodevelopment (Figure 3) in which the male brain is more sensitive than that of females to the progesterone actions, more specifically in the structures related to reproductive functions and sexual behavior in adulthood. Interestingly, the increase in the brain PR expression coincides with the period of masculinization induced by androgens during prenatal development in mice (Desroziers et al., 2017). Furthermore, PR expression in the preoptic area and the hypothalamus of developing male rats increases at E18, simultaneously to the beginning of testosterone secretion by testes. Considering that testosterone is metabolized to estradiol by the aromatase enzyme and that the ligand-bound nuclear receptor of estradiol (i.e., ERα) directly upregulates the PR expression (Weisz and Ward, 1980; Petz et al., 2004), testosterone is thought to regulate PR expression in these brain regions during rat development after its conversion into estradiol (Quadros et al., 2002c). This is supported by the decrease in PR expression levels seen in the hypothalamus of newborn male rats that were either exposed to the androstatrienedione aromatase inhibitor or whose aromatase gene expression was silenced (Quadros et al., 2002c; Brock et al., 2010). This induces an abnormal sexual male behavior during adulthood.

These studies indicate that progesterone actions and indirect PR upregulation by testosterone in sex dimorphic brain regions contribute to brain masculinization in developing males. Similarly, a study utilizing a model of masculinized female rats (following the exogenous administration of testosterone) reported that mifepristone treatment at birth blocks the masculinization effect of testosterone in the total volume of the MPN, which is larger in males than in females (Quadros et al., 2002b). Consistently, PR *knockout* male mice exhibit an impaired adult male sexual behavior (Phelps et al., 1998). Interestingly, neonatal male mice treated with the ZK137316 PR antagonist during the critical period of brain masculinization (P0–P10) display minor abnormalities related to male sexual behavior in adulthood. This is in contrast with the prepubertal treated females (P15–P25) that present an impaired lordosis behavior in adulthood (Desroziers et al., 2017), suggesting that the effects of PR activation (Figure 2) on sexual differentiation are time-dependent and sex-regulated during development.

### Neuromodulatory Actions of Progesterone During Neurodevelopment

Glutamate and GABA are the most abundant neurotransmitters in the CNS. Specifically, while glutamate is the major excitatory neurotransmitter, GABA is the main inhibitory one. Given that the CNS functionality relies on a crucial balance of neural excitation and inhibition (Petroff, 2002), progesterone can exert neuromodulatory effects through its metabolites, that can in turn interact with the specific neurotransmitter receptors coupled to the ion channels. For example, allopregnanolone is a recognized positive allosteric modulator of the ionotropic GABA-A receptors activity, which potentiates the GABA-induced chloride conductance (Figure 2) (Dorota et al., 1986; Hosie et al., 2006).

During neurodevelopment, fetal CNS progesterone and allopregnanolone levels was mainly found to increase at late stages of pregnancy in sheep (Nguyen et al., 2003; Pluchino et al., 2016). This finding was associated with the maintenance of a suppressive neural activity in the immature CNS by the GABA-A receptor modulation, thus preventing excitotoxic processes. This is consistent with the fact that the expression of GABA-A receptors in the fetal brain increases as gestation proceeds (Crossley et al., 2003; Hirst et al., 2014) and that the neurosteroid modulators of GABA-A receptors (e.g., allopregnanolone) suppress CNS activity at late gestation in fetal sheep. While GABA exerts an inhibitory activity during the fetal life in this species, it exerts an excitatory activity in the fetal brain of other species (e.g., rodents) (Bregestovski and Bernard, 2012). This indicates that allopregnanolone maintains the characteristic sleep-like behavior of the sheep fetus before birth, which is important for protecting the brain from early excitatory activity (Nicol et al., 1999). Moreover, inhibition of progesterone conversion into allopregnanolone through the administration of finasteride in the sheep fetus increases both the excitatory CNS activity and the arousal-like behavior, as found through electrocorticography, muscle electromyography and electrooculography measurements (Nicol et al., 2001). This reinforces the idea of the neuromodulatory actions of allopregnanolone in the fetal brain, which contributes to the maintenance of adequate physiological states during the prenatal life for CNS development (Crossley et al., 2003; Yawno et al., 2007).

With regards to allopregnanolone, it is relevant to mention its effects as an anxiolytic and antidepressant drug for postpartum women and rodents (Bitran et al., 1995; Osborne et al., 2017), which are mediated by the GABA-stimulated chloride ion influx (Figure 2) in the maternal brain cortex (Bitran et al., 1995). The mammalian pregnancy is characterized by an increase in the levels of progesterone and allopregnanolone present in the maternal plasma as well as in the maternal and fetal brain (Milewich et al., 1991; Osborne et al., 2017). Maternal progesterone levels begin to fall dramatically shortly before birth in most mammals to allow delivery. Indeed, the elevated levels of both progesterone and its metabolite allopregnanolone present during pregnancy progressively decay to baseline levels after delivery in humans (Tuckey, 2005). This event is related to the development of a neuropsychiatric disorder referred to as postpartum depression, which is developed by 15–20% of postpartum women (Yonkers et al., 2011; Osborne et al., 2017) and indirectly affects the healthy emotional, behavioral and cognitive development of the newborn (Kanes et al., 2017). Furthermore, women in late pregnancy with significant depression were found to have significantly lower levels of allopregnanolone than those without depressive symptoms (Hellgren et al., 2014). Interestingly, alleviation of depressive symptoms in postpartum women has been successfully achieved in phase 3 clinical trials using the drug brexanolone, an allopregnanolone analog (Meltzer-Brody et al., 2018). Overall, these studies indicate that the neuromodulatory effects of both progesterone and its metabolite allopregnanolone are relevant not only for the developing CNS but also for the adaptation of the maternal CNS to pregnancy, which in turn impacts the healthy development of the offspring.

### Neuroprotective Actions of Progesterone During Development

Protective actions of progesterone in the male and female adult brain have been widely observed both *in vitro* and *in vivo* studies. For example, progesterone has a significant role in the neurological recovery following brain and spinal cord traumatic or hypoxic-ischemic injury by reducing the oxidative damage, preserving mitochondrial functions and attenuating cell death (Deutsch et al., 2013; Andrabi et al., 2017). This supports clinical trials proposing progesterone as a therapeutic agent for the treatment of patients with traumatic brain injury (Wright et al., 2007).

The mammal neonatal CNS is still undergoing active development and exhibits processes that are essential for the establishment of the neural circuits after birth, including synaptogenesis and axon myelination (Figure 3). However, the early CNS is very susceptible to damage, such as intrauterine complications, neonatal hypoxic-ischemia derived from birth asphyxia, and acute excitotoxicity resulting from overactivation of the NMDA and AMPA receptors in neurons by the excitatory neurotransmitter glutamate. These causes of damage contribute to neuronal death and impaired neurodevelopment, which in turn induce permanent neurological deficits and neonatal death (Peterson et al., 2015; Atif et al., 2016).

As observed in the adult CNS of rats and sheep, progesterone and its metabolite allopregnanolone attenuate CNS damage when it is induced during development by regulating cell proliferation, and by reducing inflammation and apoptosis (Yawno et al., 2007, 2009; Barha et al., 2011; Peterson et al., 2015).

Treatment with progesterone for 5 days reduces the inflammation, resulting in only a few activated microglia cells in the cortex and hippocampus of neonatal rats with brain injury induced by hypoxia exposure (Peterson et al., 2015). In addition, this study indicated that progesterone-treated pups exhibited an improved motor and cognition recovery at 20 days post-injury in both genders when compared to the non-treated animals. Furthermore, this positive effect was higher in males than in females. In a similar study with neonatal mice, progesterone effects on inflammation and neuronal recovery in neonates were observed to occur without significant differences between males and females. This investigation shows that the progesterone effects can be mediated through the regulation of the secretion of pro-inflammatory cytokines as well as the upregulation of the expression of the neurotrophic factor BNDF and its specific receptor TrkB, involved in neuronal differentiation and survival (Dixon and Sherrard, 2006; Atif et al., 2016).

Using organotypic hippocampal slice cultures obtained from postnatal day 7 (P7) female and male rats, progesterone treatment was demonstrated to reduce neuronal cell death caused by oxygen/glucose deprivation. These protective effects were attenuated when the slice cultures were co-treated with mifepristone and finasteride, indicating that either progesterone itself or its metabolites exert neuroprotective effects during development (Ishihara et al., 2016). Interestingly, the disruption of allopregnanolone synthesis by finasteride administration in sheep fetuses of both genders at day 125 of gestation increased apoptosis in the CA3 and CA1 hippocampal regions, as well as in the cerebellar gray matter, accompanied by an increase in the proliferation of reactive astrocytes. These effects were reverted with the co-infusion of finasteride plus the alfaxalone allopregnanolone analog (Belelli and Lambert, 2005; Yawno et al., 2009).

### The Role of Progesterone in CNS Tumors Development

Tumorigenic processes are often observed from the developmental biology perspective, given that they frequently recapitulate molecular and cellular events carried out by stem cells that shape the organism during development. Cancer is considered as an abnormal reactivation of a developmental program, in which the tumor, and all its intracellular heterogeneity, can be visualized as a developing organ (Wechsler-Reya and Scott, 2001; Laug et al., 2018).

Gliomas are the most prevalent and malignant type of tumors occurring in the human CNS and they arise from the uncontrolled proliferation of glia, progenitor glial cells or cancer stem cells (Louis et al., 2016). Astrocytomas are the most frequent type of glioma (Ostrom et al., 2017), which are classified by the World Health Organization (WHO) from grade I to IV, according to their histopathological and malignant characteristics. Specifically, grade IV, which is also referred to as glioblastoma multiforme (GBM), is the most malignant type of astrocytoma (Louis et al., 2016; Ostrom et al., 2017) due to its high capacity to proliferate and invade the normal brain tissue (Stupp et al., 2005). Several factors within the CNS contribute to GBM tumorigenesis and, in recent years, sex hormones, particularly progesterone, were proposed as the factors involved in GBM tumor progression. The first insights into the involvement of progesterone in the development of GBM emerged from the observation that human glioblastoma cells express the two PR isoforms (González-Agüero et al., 2007), suggesting a function for these receptors in this type of cancer. Furthermore, studies *in vitro* demonstrated that progesterone present at the physiological levels (10 nM) increases the content of many proteins, including SRC-1/3, p-Akt, and p-Src (Hernández-Hernández et al., 2010; González-Orozco et al., 2018), in GBM cells. In addition, the same concentration of progesterone was found to upregulate the expression of key genes for cell proliferation, neovascularization, and tumor growth (e.g., the vascular endothelial growth factor, the receptor for the epidermal growth factor and cyclin D1) (Hernández-Hernández et al., 2012). Similarly, progesterone and allopregnanolone increase cell proliferation, migration and invasion of GBM cells *in vitro* (González-Agüero et al., 2007; Piña-Medina et al., 2016; Zamora-Sánchez et al., 2017). Moreover, progesterone also promotes GBM tumor growth and infiltration in rats’ cerebral cortex in a xenograft model (Germán-Castelán et al., 2014, 2016), effects which are mainly mediated by the PR, given that they are partially blocked in GBM cells following the administration of mifepristone (González-Agüero et al., 2007; Germán-Castelán et al., 2014; Piña-Medina et al., 2016). This partial inhibition indicated the implication of the non-classical progesterone mechanisms of action. Recent studies describe that GBM cells express both mPRα and mPRβ, and that the specific activation of mPRα by the selective agonist Org OD 02-0 induces cell proliferation and invasion (Valadez-Cosmes et al., 2015; González-Orozco et al., 2018). Similar effects are seen when the PR is activated via ligand-independent activation by protein kinase C (González-Arenas et al., 2015; Marquina-Sánchez et al., 2017), suggesting an interplay between the classical and non-classical mechanisms of progesterone action in GBM tumors progression.

In contrast, high concentrations of progesterone (20–80 μM) were reported to reduce GMB tumor growth in a xenograft mouse model by increasing apoptosis and inhibiting cell proliferation, which was accompanied by an improvement in the survival rate (Atif et al., 2015). These results suggest that progesterone promotes GBM tumor progression when present at the physiological levels, whereas it has opposite effects at high concentrations, inhibiting GBM growth.

## Conclusion

Progesterone actions extend beyond the regulation of brain functions associated with reproduction. In fact, its physiological actions extend to several non-reproductive functions in the CNS of both male and female mammals from early prenatal life, by regulating the fundamental molecular and cellular processes underlying its proper development.

Understanding the precise role of progesterone and other steroid hormones is relevant to the overall comprehension of the nervous system development and its subsequent normal functioning during life. Important clinical advances were made, including the implementation of progesterone treatment in pre-clinical phase 2 trials to assess its efficacy as a neuroprotector in patients with traumatic brain injury and the recent success in pre-clinical phase 3 trials to treat depression in postpartum women by using the analogous compounds of allopregnanolone (Schumacher et al., 2012; Meltzer-Brody et al., 2018).

## Perspectives

The study of sex steroid hormone actions during the development of the nervous system in the prenatal and early postnatal life is yet to be widely explored. Therefore, this field implies promising research that will provide us with a better understanding of the molecular and cellular processes underlying the mechanisms that shape this system. In fact, the knowledge of the mechanisms of action of sex hormones, including progesterone, is essential to fully understand the critical processes that occur in neurodevelopment (e.g., neurogenesis, gliogenesis, myelination, neuroprotection, neural circuit maturation, and brain sexual differentiation). Once the precise involvement of sex hormones in neurodevelopment is known, this knowledge could be translated to attempting to reverse specific pathologies related to the cited processes during postnatal life. Understanding the mechanisms of progesterone and its metabolites, the types of cellular receptors that are activated, the signaling pathways involved, the regulated genes, and the cytokines secreted by the effects of these steroids in the nervous tissue, could be advantageous in clinical therapies to: (1) reduce premature infants’ CNS susceptibility to injury, which could in turn allow the normal behavioral and cognitive development of the infant; (2) optimize the therapeutic strategies that are already in use to alleviate the depression and anxiety symptoms seen in postpartum women; and (3) attenuate the damage in demyelinating lesions or diseases. Finally, the understanding of progesterone actions in brain tumors development may also be useful to elaborate more effective clinical approaches aimed at stopping the progression of such neoplasms in patients.

## Author Contributions

Both authors have contributed equally to the elaboration of the manuscript.

## Conflict of Interest Statement

The authors declare that the research was conducted in the absence of any commercial or financial relationships that could be construed as a potential conflict of interest.
